# Role of the caspase-8/RIPK3 axis in Alzheimer’s disease pathogenesis and A**β**-induced NLRP3 inflammasome activation

**DOI:** 10.1172/jci.insight.157433

**Published:** 2023-02-08

**Authors:** Sushanth Kumar, Sakar Budhathoki, Christopher B. Oliveira, August D. Kahle, O. Yipkin Calhan, John R. Lukens, Christopher D. Deppmann

**Affiliations:** 1 Department of Biology and; 2 Neuroscience Graduate Program, School of Medicine, and; 3 Center for Brain Immunology and Glia (BIG), Department of Neuroscience, School of Medicine, University of Virginia, Charlottesville, Virginia, USA.

**Keywords:** Inflammation, Neuroscience, Alzheimer disease, Apoptosis pathways, Innate immunity

## Abstract

The molecular mediators of cell death and inflammation in Alzheimer’s disease (AD) have yet to be fully elucidated. Caspase-8 is a critical regulator of several cell death and inflammatory pathways; however, its role in AD pathogenesis has not yet been examined in detail. In the absence of caspase-8, mice are embryonic lethal due to excessive receptor interacting protein kinase 3–dependent (RIPK3-dependent) necroptosis. Compound RIPK3 and caspase-8 mutants rescue embryonic lethality, which we leveraged to examine the roles of these pathways in an amyloid β–mediated (Aβ-mediated) mouse model of AD. We found that combined deletion of caspase-8 and RIPK3, but not RIPK3 alone, led to diminished Aβ deposition and microgliosis in the mouse model of AD carrying human presenilin 1 and amyloid precursor protein with 5 familial AD mutations (5xFAD). Despite its well-known role in cell death, caspase-8 did not appear to affect cell loss in the 5xFAD model. In contrast, we found that caspase-8 was a critical regulator of Aβ-driven inflammasome gene expression and IL-1β release. Interestingly, loss of RIPK3 had only a modest effect on disease progression, suggesting that inhibition of necroptosis or RIPK3-mediated cytokine pathways is not critical during midstages of Aβ amyloidosis. These findings suggest that therapeutics targeting caspase-8 may represent a novel strategy to limit Aβ amyloidosis and neuroinflammation in AD.

## Introduction

Alzheimer’s disease (AD) is a progressive neurodegenerative disease that is characterized by the accumulation of amyloid β (Aβ) and tau. The buildup of these species and other neurotoxic factors is associated with an upregulation of pro-inflammatory cytokine production and cell death signaling (1). Initially, these responses were thought to be beneficial and aid in the clearance of harmful aggregates (2, 3). However, in AD, these physiologic responses shift to chronic activation, which can give rise to deleterious neuroinflammation, neuronal loss, and cognitive decline. Although recent single-cell transcriptomic studies have provided some insights into how cells transition from a healthy to diseased state during AD, as well as pathways that may drive pathology, we still lack full knowledge of the molecular mediators responsible for propagating inflammation and cell death in AD (4–6).

Caspase-8 is an essential regulator of several cell death and inflammatory pathways. Caspase-8 was first described as a key component of the extrinsic apoptotic pathway, which is mediated through death receptor–ligand interactions (e.g., TNF receptor superfamily [TNFRSF] members). Upon death receptor activation, adapter proteins and procaspase-8 are recruited, promoting the dimerization and autoprocessing of procaspase-8 to its active form, caspase-8, which then proceeds to execute apoptosis via caspase-3 activation (7). Upregulation of death receptors has been observed in patients with AD (8, 9). Early studies have also demonstrated the presence of active forms of caspase-3 and caspase-8 in the brains of patients with AD (10, 11). However, whether extrinsic apoptotic programs are required for the cell death associated with AD has not been fully examined to date, to our knowledge.

In addition to promoting apoptosis, caspase-8 is an important negative regulator of receptor interacting protein kinase 1–receptor interacting protein kinase 3–mediated (RIPK1-RIPK3–mediated) necroptosis, which is an inflammatory form of cell death. The role that caspase-8 plays in curbing necroptosis is especially important during development, when the deletion of caspase-8 alone results in excessive RIPK1-RIPK3–driven necroptosis and leads to embryonic lethality in caspase-8–deficient mice (12–14). Interestingly, various components of the necroptotic signaling pathway including RIPK1 and RIPK3 have been implicated in several neurodegenerative conditions (14–17). Therefore, the possibility exists that RIPK3 deletion alone or in combination with caspase-8 deletion also affects Alzheimer’s-related disease pathogenesis (18). *Casp8^–/–^Ripk3^–/–^* mice are deficient in both extrinsic apoptosis and necroptosis and are viable, which provides us an opportunity to study the contributions of these 2 cell death and signaling pathways in AD (19–21).

Beyond regulating cell death, caspase-8 also acts as a key mediator of inflammatory cytokine production. After death receptor or TLR activation, caspase-8 can promote NF-κB–mediated cytokine production (e.g., TNF-α and IL-6) (22, 23). Caspase-8 can also promote inflammation through its regulation of NLRP3 inflammasome signaling (24–26). The NLRP3 inflammasome is a multiprotein complex that plays an essential role in innate immunity. Activation of the inflammasome leads to release of the pro-inflammatory cytokines IL-1β and IL-18, as well as the execution of a gasdermin D–driven form of cell death known as pyroptosis (27). An additional component of inflammasome activation is the formation and release of the adapter protein apoptosis-associated speck-like protein containing a CARD (ASC). Previous reports have shown that released ASC specks can bind to Aβ, accelerate its aggregation, and increase its toxicity to bystander cells. Moreover, inhibition of ASC speck formation either genetically or pharmacologically has been shown to blunt pathology in multiple AD mouse models (28–32). To date, to our knowledge, the role of caspase-8 in AD-associated inflammasome function has not been investigated.

In this study, we set out to determine the contributions of caspase-8 and RIPK3 in Aβ amyloidosis. We demonstrate here that combined loss of caspase-8 and RIPK3 in mice carrying human presenilin 1 and amyloid precursor protein (APP) with 5 familial AD mutations (5xFAD mice) decreases overall AD pathology. More specifically, we observed that combined loss of caspase-8 and RIPK3 reduced Aβ deposition and microgliosis in the 5xFAD mouse model of AD. Although we did not observe a role for caspase-8 and/or RIPK3 in regulating neuronal loss, we did find that the caspase-8/RIPK3 signaling axis regulates NLRP3 inflammasome signaling and IL-1β secretion in response to Aβ amyloidosis.

## Results

### Induction of caspase-8 and RIPK3 expression in AD.

**Figure 1:**
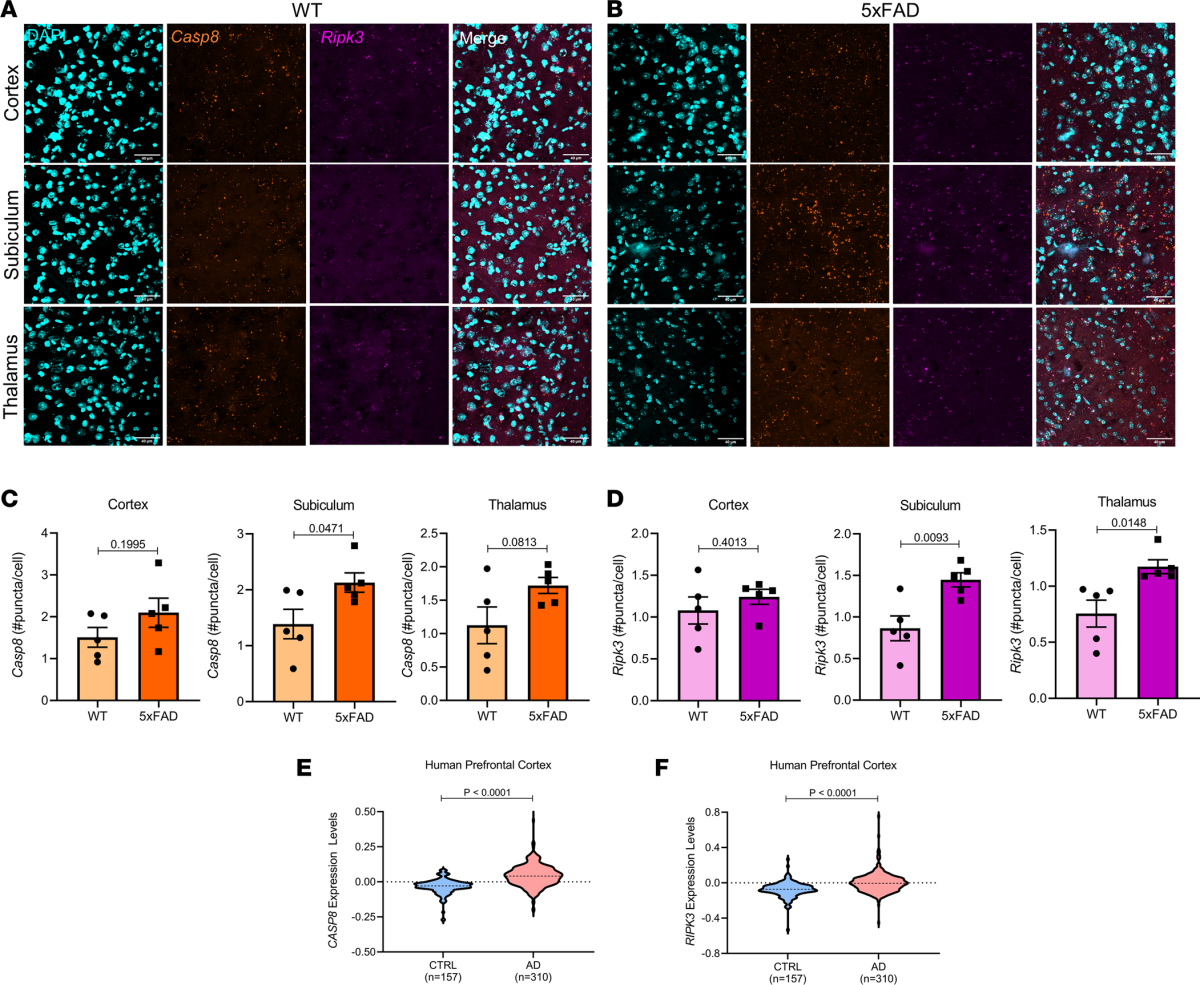
Induction of caspase-8 and RIPK3 expression in AD. (**A** and **B**) Representative images of the cortex, subiculum, and thalamus from 5-month-old WT and 5xFAD mice stained with *Casp8* (orange) and *Ripk3* (magenta) RNAscope probes. Scale bars: 40 μm. (**C**) Quantification of *Casp8* staining. (**D**) Quantification of *Ripk3* staining (*n* = 5 5xFAD mice, *n* = 5 WT mice). (**E** and **F**) *CASP8* and *RIPK3* expression data obtained from a human transcriptomic data set (GSE33000) of dorsolateral prefrontal cortex tissue from 157 patients without dementia and 310 patients with AD. (**C**–**F**) Data were analyzed by Student’s *t* test. Data reported as mean ± SEM (**C** and **D**) or as violin plots (**E** and **F**). Ctrl, control.

To examine whether caspase-8 and/or RIPK3 are altered in AD, we performed RNAscope to evaluate mRNA expression in 5-month-old WT and 5xFAD mice. *Ripk3* expression was increased in both the subiculum and thalamus, whereas *Casp8* expression was only significantly elevated in the thalamus of 5xFAD mice relative to WT controls. In the cortex, no significant differences were observed in *Casp8* or *Ripk3* between 5xFAD and WT mice (Figure 1, A–D). In addition, we also analyzed a published transcriptomic data set (NCBI Gene Expression Omnibus accession number GSE33000) from human prefrontal cortex samples, which revealed increased expression of both *CASP8* and *RIPK3* in patients with AD (Figure 1, E and F). Overall, these findings suggest there may be a role for extrinsic apoptotic and necroptotic pathways in both the 5xFAD mouse model and human AD brain.

### Combined loss of caspase-8 and RIPK3 reduces Aβ amyloidosis in 5xFAD mice.

To investigate a role for caspase-8 and RIPK3 in AD-related pathogenesis, we generated compound mutants between 5xFAD mice and *Caspase8^–/–^* and/or *Ripk3^–/–^* mice. Because of the early lethality of caspase-8 single-KO mice, 5xFAD *Casp8^–/–^* *Ripk3^–/–^* (i.e., double KO [DKO]) mice were compared with age- and sex-matched 5xFAD and 5xFAD *Ripk3^–/–^* littermate controls to evaluate the role of caspase-8 in AD pathogenesis.

Before tissue collection, 5xFAD, 5xFAD *Ripk3^–/–^*, and 5xFAD-DKO mice were aged to 5 months. We first assessed deposition of Aβ by staining with thioflavin S (ThioS), which marks plaques. In 5xFAD mice, deposition begins in the subiculum before spreading to overlying cortical regions. One of the first cortical regions affected in the 5xFAD line is the retrosplenial cortex (RS). Interestingly, we found that combined deletion of caspase-8 and RIPK3 led to a marked reduction in the number of Aβ plaque deposits in the RS. In contrast, loss of RIPK3 alone did not significantly reduce Aβ, as measured by ThioS staining (Figure 1, A and B). We observed a similar degree of Aβ reduction in 5xFAD-DKO mice when quantifying ThioS^+^ puncta in the subiculum of the hippocampus at 3 and 5 months (Supplemental Figure 1, A, C, E, and G; supplemental material available online with this article; https://doi.org/10.1172/jci.insight.157433DS1). Consistent with findings of previous studies, we did not yet observe appreciable levels of Aβ deposition in the cortex of 3-month-old 5xFAD mice (33).

**Figure 2:**
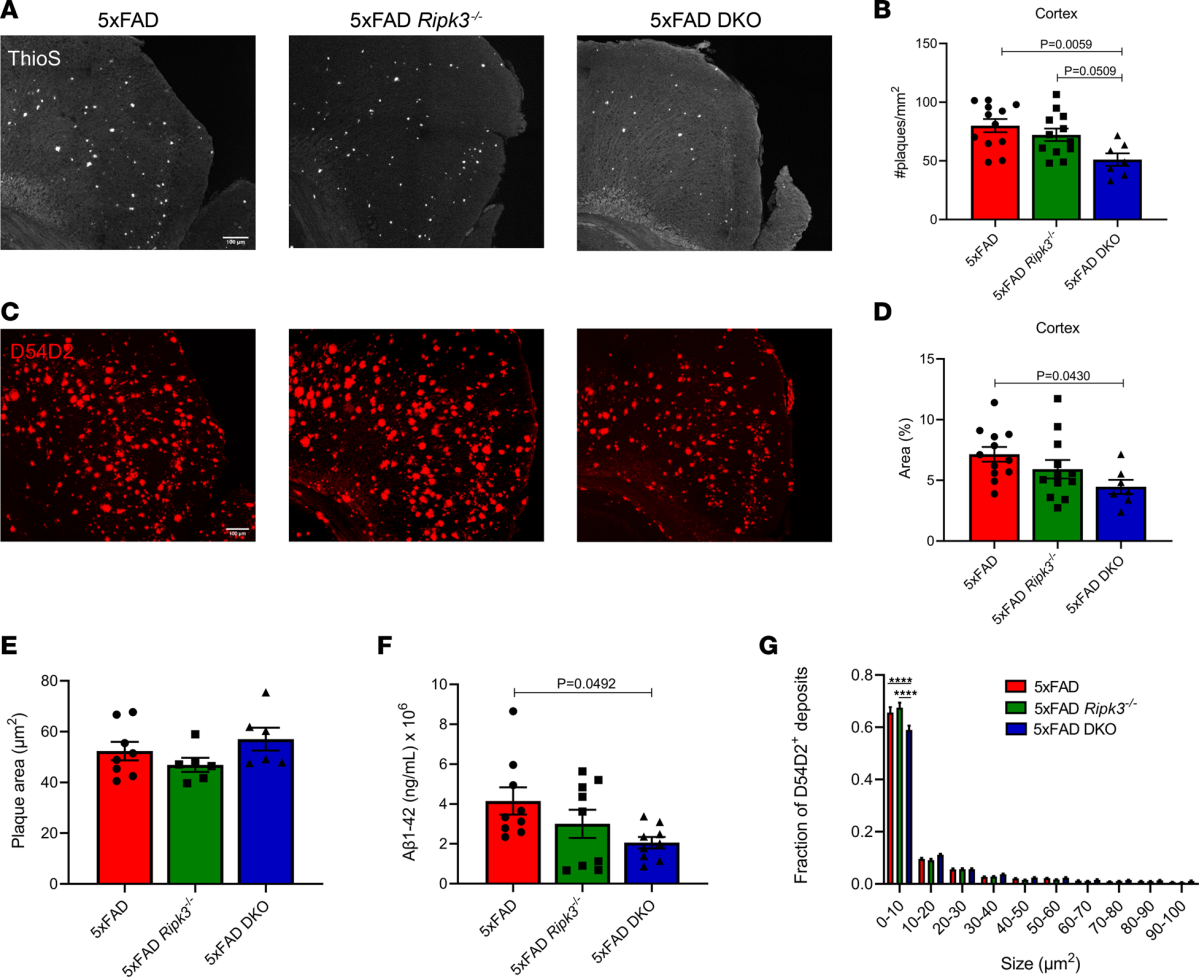
The caspase-8/RIPK3 axis promotes Aβ amyloidosis in 5xFAD mice. (**A** and **C**) Representative IHC images of the RS taken at ×20 original magnification (*n* = 12 5xFAD mice, *n* = 12 5xFAD *Ripk3^–/–^* mice, and *n* = 7 5xFAD-DKO mice). Scale bar: 100 μm. (**A**) ThioS staining in the RS. (**B**) Quantification of ThioS staining. Each data point is an average of 2–3 different sections per mouse. (**C**) D54D2 staining in the RS. (**D**) Quantification of D54D2 staining. Each data point is an average of 2–3 different sections per mouse. (**E**) Aβ1-42 ELISA for guanidine-soluble fraction from cortices of 5-month-old mice (*n* = 9 5xFAD mice, *n* = 9 5xFAD *Ripk3^–/–^* mice, *n* = 9 5xFAD-DKO mice). (**F**) Quantification of the mean plaque size from D54D2 staining in RS (*n* = 8 5xFAD mice, *n* = 6 5xFAD *Ripk3^–/–^* mice, and *n* = 6 5xFAD-DKO mice). (**G**) Fraction of D54D2 deposits falling within designated bins. Analysis was carried out in the RS for deposits less than 100 μm^2^ (*n* = 8 5xFAD mice, *n* = 6 5xFAD *Ripk3^–/–^* mice, and *n* = 6 5xFAD-DKO mice). (**A**–**F**) Data were analyzed by 1-way ANOVA followed by Tukey’s post hoc test. (**G**) Data were analyzed by 2-way ANOVA followed by Tukey’s post hoc test. All *n* values refer to the number of mice used, and error bars indicate SEM. ****P < 0.0001.

In addition to ThioS, we stained with the D54D2 Ab, which captures both dense, core Aβ plaques in addition to smaller, oligomeric species. Loss of caspase-8 along with RIPK3 mitigated D54D2 deposition in the RS compared with the RS of 5xFAD mice (Figure 2, C and D). In contrast, depletion of RIPK3 alone on the 5xFAD background only resulted in a slight trend toward decreased D54D2 staining in the RS; however, this did not reach statistical significance (Figure 2, C and D). Interestingly, we did not observe significant changes to subicular D54D2 deposition at either 3 or 5 months (Supplemental Figure 1, B, D, F, and H). Consistent with our cortical D54D2 data, we also observed that the levels of cortical guanidine-soluble Aβ1-42 were markedly reduced in 5xFAD-DKO versus 5xFAD mice via ELISA (Figure 2E). Plaque parameters such as area, volume, intensity, and sphericity (a measure of plaque compactness) were unchanged between genotypes (Figure 2F and Supplemental Figure 2). D54D2 staining revealed much more heterogeneity in plaque sizes. Although average plaque size was similar between genotypes, combined loss of caspase-8 and RIPK3 led to a significant reduction in the number of smaller (0–10 μm^2^) D54D2^+^ deposits (Figure 2G). Overall, these data suggest that caspase-8 contributes to amyloid deposition in the 5xFAD model.

### Reduced microgliosis with loss of caspase-8 and RIPK3 in 5xFAD mice.

**Figure 3:**
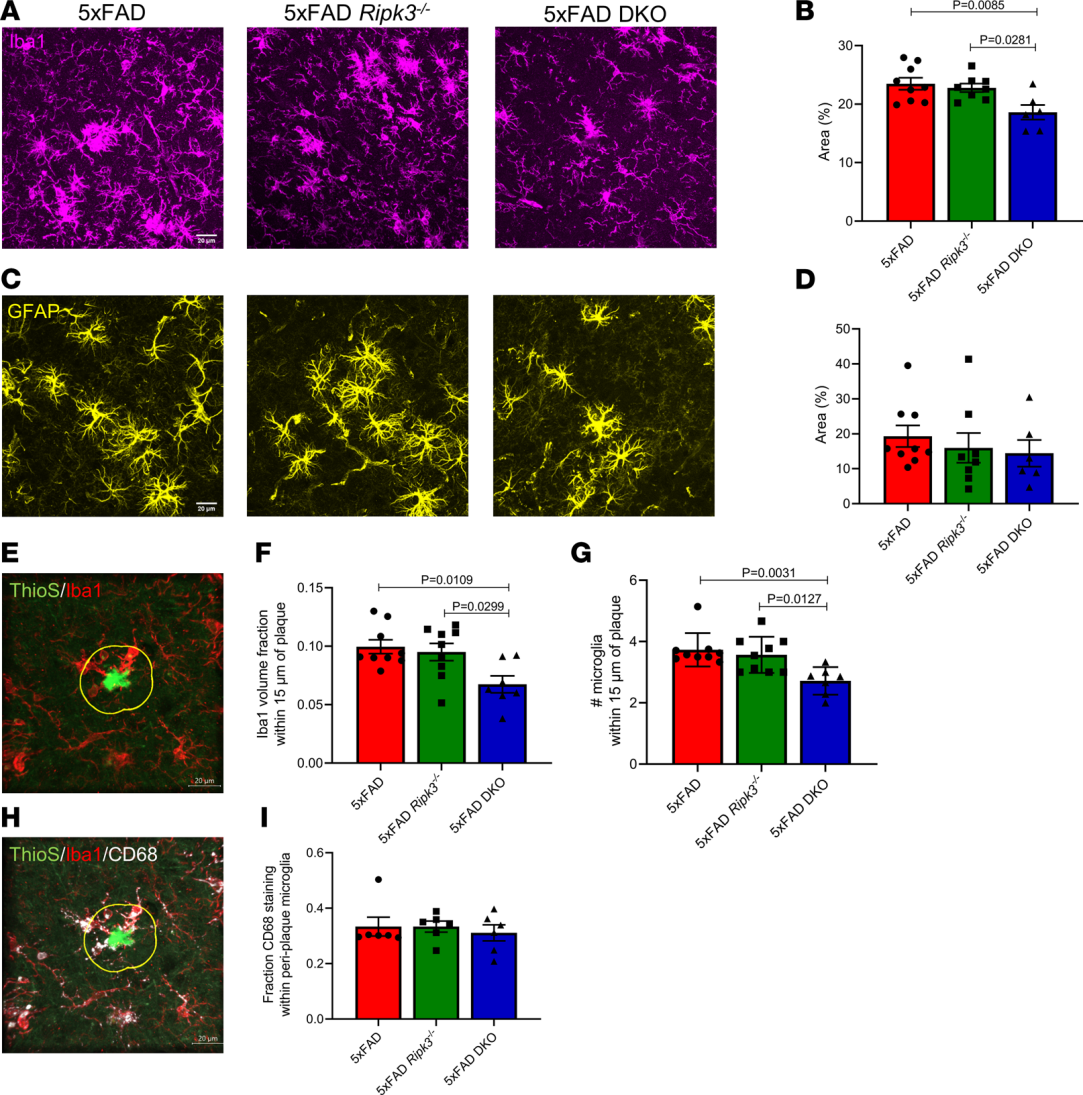
Reduced microgliosis with loss of caspase-8 and RIPK3 in 5xFAD mice. (**A** and **C**) Representative IHC images of the cortex taken at ×40 original magnification (*n* = 9 5xFAD mice, *n* = 8 5xFAD *Ripk3^–/–^* mice, and *n* = 6 5xFAD-DKO mice). (**A**) Iba1 staining. (**B**) Quantification of Iba1 staining. Each data point is an average of 10 different fields of view taken throughout the cortex spanning 2 different sections per mouse. (**C**) GFAP staining. (**D**) Quantification of GFAP staining. Each data point is an average of 10 different fields of view taken throughout the cortex spanning 2 different sections per mouse. (**E**) Image of cortical Iba1^+^ microglia (red) surrounding ThioS^+^ plaque (green) within a 15 μm barrier (yellow) measured from plaque surface taken at ×40 original magnification. (**F**) Volume of Iba1 staining within the 15 μm barrier was quantified and divided by total barrier volume. (**G**) Number of microglia as identified by Spots function on Imaris 9.7.2 were counted within the barrier. (**F** and **G**) Results from 6–10 plaques were averaged per data point (*n* = 9 5xFAD mice, *n* = 9 5xFAD *Ripk3^–/–^* mice, and *n* = 7 5xFAD-DKO mice). (**H**) Image of microglia (red) costained with CD68 (white) surrounding ThioS^+^ plaque (green) within a 15 μm barrier (yellow) measured from plaque surface taken at ×40 original magnification. (**I**) Quantification of cortical microglial CD68 staining within a 15 μm periplaque barrier. Results from 6–10 plaques were averaged per data point. Data were analyzed by 1-way ANOVA followed by Tukey’s post hoc test. All *n* values refer to the number of mice used, and error bars indicate SEM. Scale bars: 20 μm.

Chronic deposition of Aβ leads to a persistent glial response that promotes neuroinflammation. Moreover, excessive microglial and astrocyte engagement is thought to promote further Aβ deposition (34). Consistent with findings of previous studies, 5xFAD mice had elevated ionized calcium-binding adapter molecule 1 (Iba1) immunoreactivity compared with that of nontransgenic littermates (Figure 3, A and B, and Supplemental Figure 4, A and C) (35). We found that 5xFAD-DKO mice had significantly reduced cortical Iba1 staining compared with both 5xFAD and 5xFAD *Ripk3^–/–^* mice. A similar reduction was also seen in the subiculum of 3-month-old mice (Supplemental Figure 3). Microgliosis was largely absent in the cortex of 3-month-old mice, likely because of lack of cortical amyloid pathology at this early time point (data not shown).

To rule out the possibility of DKO microglia undergoing increased cell death, we performed TUNEL staining and observed negligible microglial TUNEL positivity across our different genotypes (Supplemental Figure 5). We also investigated astrocyte reactivity but did not observe any significant differences in glial fibrillary acidic protein (GFAP) staining across genotypes (Figure 3, C and D). It should be noted that outside of the 5xFAD background, loss of caspase-8 and/or RIPK3 did not alter baseline Iba1 or GFAP staining (Supplemental Figure 4, A–D).

In AD, microglia form barriers around plaques, and this is critical for minimizing neuronal damage but may also contribute to overall gliosis (36–38). We analyzed cortical microglia within 15 μm of ThioS^+^ plaques and found that combined loss of caspase-8 and RIPK3 decreased both the total number of Iba1^+^ cells and overall Iba1 staining in periplaque regions (Figure 3, E–G). Because we observed an overall decrease in Aβ amyloid load in 5xFAD-DKO mice (Figure 2, A–E), we next wanted to determine if the caspase-8/RIPK3 axis regulates microglial phagocytosis of Aβ. To this end, we stained for CD68, a phagolysosomal marker, and focused our attention on plaque-associated microglia. We observed that periplaque microglia in all genotypes had a similar degree of CD68 expression (Figure 3, H and I). This finding suggests that microglial phagocytic capacity in 5-month-old 5xFAD mice is not likely significantly altered by deletion of RIPK3 alone or in combination with caspase-8.

### Reduction of microglial activation with loss of caspase-8 and RIPK3 in 5xFAD mice.

**Figure 4:**
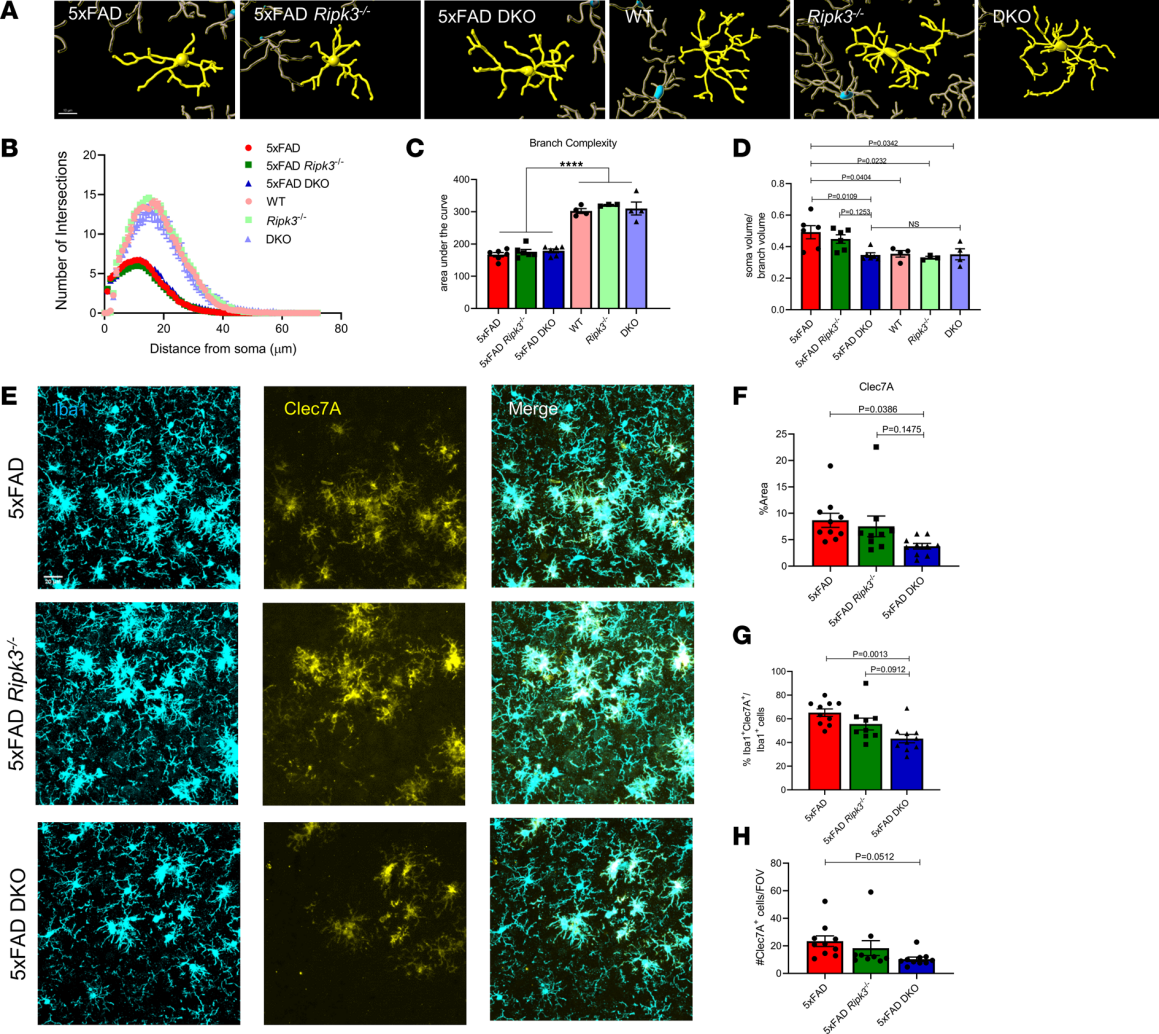
Reduction of microglial activation with loss of caspase-8 and RIPK3 in 5xFAD mice. (**A**) Representative reconstructions of Iba1^+^ cells. Scale bar: 10 μm. (**B**) Sholl analysis for nonplaque–associated microglia. Each data point represents the number of Iba1^+^ branches intersecting with a radius of 0–80 μm from the soma, calculated by the average of 70–80 microglia per group (*n* = 6 mice/group). (**C**) Quantification of the area under the curve for the microglial Sholl analysis data. Each data point represents a single mouse. ****P < 0.0001. (**D**) Ratio of the microglia soma volume (measured using Surfaces feature in Imaris 9.7.2) to the branch volume. (**A**–**D**) (*n* = 6 5xFAD mice, *n* = 6 5xFAD *Ripk3^–/–^* mice, *n* = 6 5xFAD-DKO mice, *n* = 4 WT mice, *n* = 3 *Ripk3^–/–^* mice, *n* = 4 DKO mice). (**E**) Representative IHC images of cortex taken at ×40 original magnification stained for Iba1 (cyan) and Clec7A (yellow) (*n* = 10 5xFAD mice, *n* = 9 5xFAD *Ripk3^–/–^* mice, and *n* = 10 5xFAD-DKO mice). (**F**) Quantification of percent area coverage of Clec7A staining. (**G**) Quantification of the number of Clec7A^+^ cells per field of view (FOV). (**H**) Percentage of Iba1^+^Clec7A^+^ double-positive cells per FOV. Data were analyzed by 1-way ANOVA followed by Tukey’s post hoc test. All *n* values refer to the number of mice used, and error bars indicate SEM.

To evaluate if combined loss of caspase-8 and RIPK3 influences microglial morphology, we next performed Sholl analysis. We did not observe any differences in overall microglial branch complexity in the cortex across the genotypes (Figure 4, A–D). We did, however, find that the combined loss of caspase-8 and RIPK3 in 5xFAD mice led to a decrease in the ratio of soma to branch volume (Figure 4D). As AD progresses, microglia undergo a shift in their transcriptome, which is often referred to as a disease-associated microglial (DAM) signature (5, 6). One of the genes that shows a marked upregulation in the DAM population is the pattern recognition receptor Clec7A, which is largely absent in homeostatic microglia. We stained for Clec7A and observed that 5xFAD DKO microglia exhibit significantly reduced Clec7A staining (Figure 4, E–H). Overall, these data suggest that combined loss of caspase-8 and RIPK3 leads to an overall decrease in microgliosis in response to Aβ amyloidosis.

### Loss of caspase-8 and/or RIPK3 does not affect attrition of cortical layer V neurons in 5xFAD mice.

**Figure 5:**
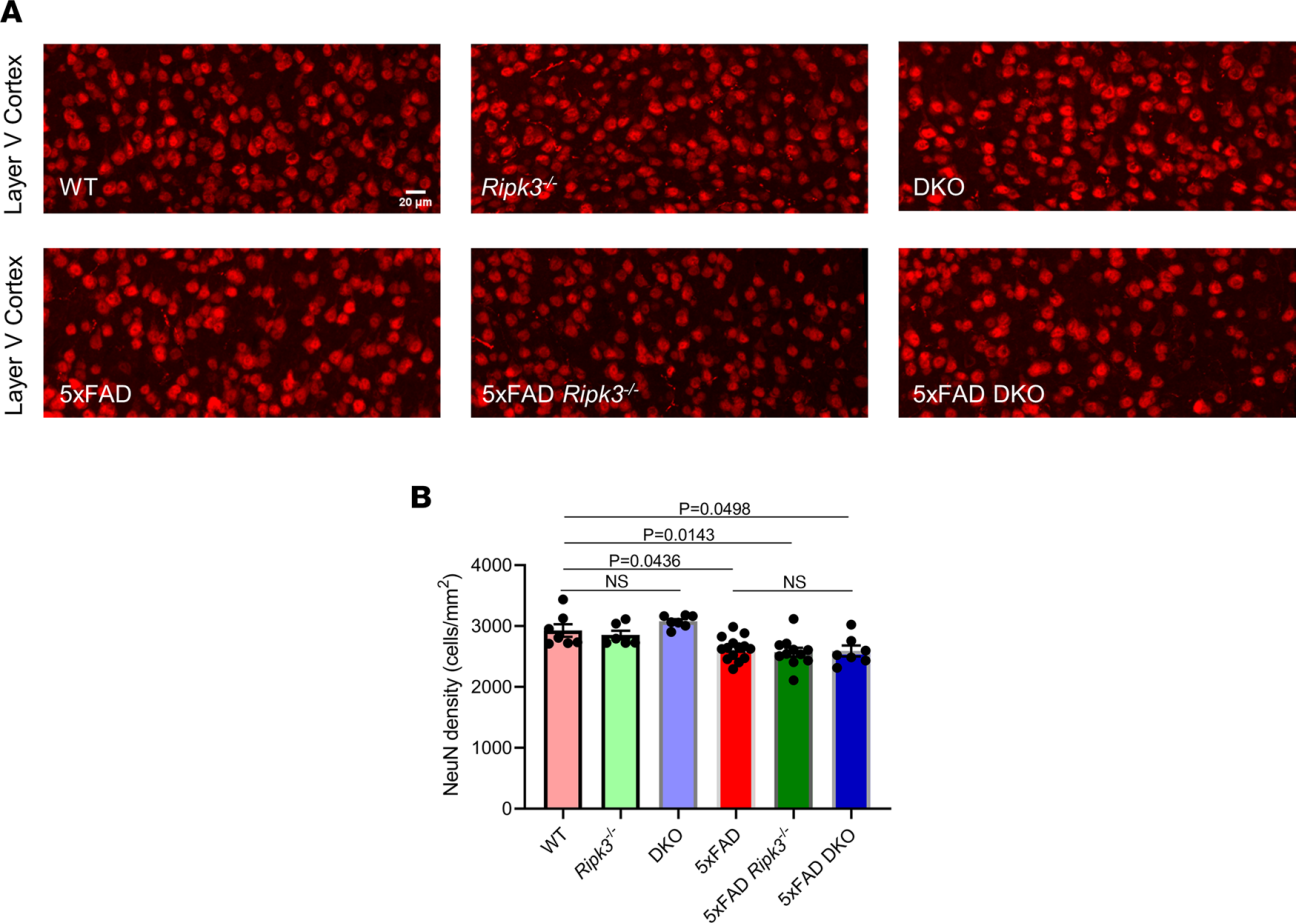
Loss of caspase-8 and/or RIPK3 does not alter cortical layer V neuron density in 5xFAD mice. (**A**) Representative IHC images of cortical layer V stained for NeuN taken at ×20 original magnification (*n* = 7 WT mice, *n* = 6 *Ripk3^–/–^* mice, *n* = 7 DKO mice, *n* = 14 5xFAD mice, *n* = 11 5xFAD *Ripk3^–/–^* mice, and *n* = 7 5xFAD-DKO mice). (**B**) Quantification of NeuN staining. Data were analyzed by 1-way ANOVA followed by Tukey’s post hoc test. Data expressed as mean ± SEM.

Cell death is a key driver of neurodegenerative disease. Therefore, it is possible that changes in the amount of cell death upon elimination of caspase-8 and/or RIPK3 could account for some of the phenotypes we observed. Various groups have demonstrated that 5xFAD mice display age-dependent neuronal loss, especially in cortical layer V (39, 40). To evaluate neuronal loss, we counted NeuN^+^ cells in layer V of the cortex. Layer V neurons were identified using the layer V/VI marker chicken ovalbumin upstream promoter transcription factor interacting protein 2 (Ctip2) (Supplemental Figure 6A). Significant reductions in NeuN cell density were observed in 5xFAD mice relative to nontransgenic WT controls. However, we did not observe significant differences in neuronal counts across 5-month-old 5xFAD, 5xFAD *Ripk3^–/–^*, and 5xFAD-DKO mice. Corresponding non-5xFAD control mice also showed no differences in NeuN density (Figure 5). Quantification of Ctip2 density revealed a similar pattern to the analysis from NeuN staining (Supplemental Figure 6, A and B). These data suggest that deletion of RIPK3 alone or combination with caspase-8 does not appreciably affect neuronal cell death in cortical layer V.

### The caspase-8/RIPK3 axis regulates NLRP3 inflammasome priming and activation in response to Aβ.

If not by cell death, how does caspase-8 affect AD progression? We speculated that caspase-8 may regulate inflammatory cytokine production in response to Aβ. Indeed, authors of recent reports in other models of disease have identified caspase-8 as a key regulator of the NLRP3 inflammasome, where caspase-8 has been shown to influence both the priming and activation steps of inflammasome signaling (24, 25, 41). Our results suggest a role for caspase-8 in regulating AD-associated gliosis; however, the question remains whether this is through inflammasome activation or an alternative mechanism.

**Figure 6:**
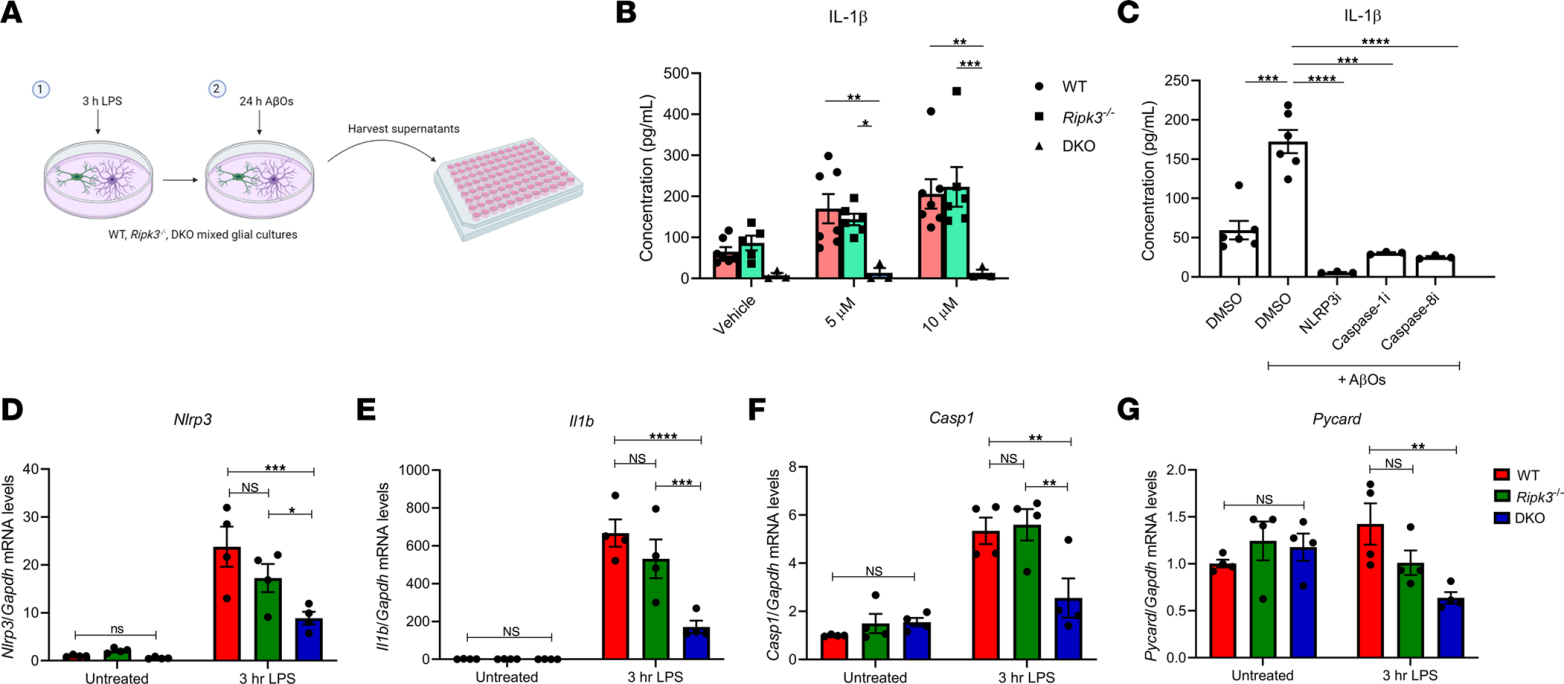
Caspase-8 promotes Aβ-induced IL-1β release from mixed astrocyte–microglia cultures. (**A**) Schematic for treatment paradigm. Mixed astrocyte–microglia cultures were primed for 3 hours with 500 ng/mL LPS, then treated for 24 hours with oAβs. Supernatants were then harvested and evaluated for IL-1β release. (**B**) Supernatants were collected from WT, *Ripk3^–/–^*, and DKO mixed glial cultures after 24 hours of treatment with either vehicle, 5 μM, or 10 μM oAβs, and evaluated for IL-1β release via ELISA. 5 μM: WT versus DKO **P = 0.0087, and *Ripk3^–/–^* versus DKO *P = 0.0381; 10 μM: WT versus DKO **P = 0.0012, and *Ripk3^–/–^* versus DKO ***P = 0.006. (**C**) WT mixed glial cultures were treated with oAβs either in the presence of an NLRP3 inhibitor (NLRP3i) (MCC950, used at 1 μM), a caspase-1 inhibitor (caspase-1i) (Ac-YVAD-cmk, used at 10 μM), or a caspase-8 inhibitor (caspase-8i) (zIETD-fmk, used at 10 μM). DMSO versus DMSO (+ oAβs), ***P < 0.001; DMSO (+ oAβs) versus NLRP3i, ****P < 0.0001; DMSO (+ oAβs) versus caspase-1i, ***P < 0.001; DMSO (+ oAβs) versus caspase-8i, ****P < 0.0001. (**D**–**G**) Mixed-culture gene transcript levels for *Nlrp3*, *Il1b*, *Casp1*, and *Pycard* before and after 3-hour LPS priming (*n* = 4 WT mice, *n* = 4 *Ripk3^–/–^* mice, and *n* = 4 DKO mice). *P < 0.05, **P < 0.01, ***P < 0.001, ****P < 0.0001. (**B**) Data were analyzed by 2-way ANOVA followed by Tukey’s post hoc test. (**C**–**G**) Data were analyzed by 1-way ANOVA followed by Tukey’s post hoc test. Data are from at least 3 independent experiments and expressed as mean ± SEM.

We first sought to probe this question in an in vitro setting. To investigate AD-associated inflammasome signaling and IL-1β production, we generated mixed astrocyte–microglia cultures from P0 to P2 and challenged them with synthetic Aβ oligomer preparations (Figure 6A). Consistent with findings of prior studies, we observed that Aβ can activate the NLRP3 inflammasome, resulting in IL-1β release (Figure 6, B and C) (28, 30, 42). Inhibiting either NLRP3 (MCC950) or caspase-1 (Ac-YVAD-cmk) led to a reduction in the secretion of IL-1β by mixed glial cultures that were stimulated with Aβ oligomers (Figure 6C). IL-1β release was also significantly dampened in DKO mixed glial cultures that were treated with Aβ oligomers (Figure 6B). Similarly, pretreatment with a caspase-8 enzymatic inhibitor (zIETD-fmk) resulted in significant reductions to IL-1β release in response to Aβ oligomers (Figure 6C). To facilitate IL-1β release, cultures were pretreated with LPS for 3 hours (Figure 6A). Previous work has implicated caspase-8 as important in the transcriptional upregulation of inflammasome components (24). After LPS priming, we observed significant increases in *Nlrp3*, *Il1b*, and *Casp1* transcripts in both WT and *Ripk3^–/–^* mixed glial cultures. DKO cultures, however, showed a marked blunting. Levels of *Pycard* (a gene encoding for ASC) did not show increases after LPS priming, but we did observe that primed DKO cultures exhibited a downregulation of *Pycard* expression after LPS stimulation (Figure 6, D–G).

**Figure 7:**
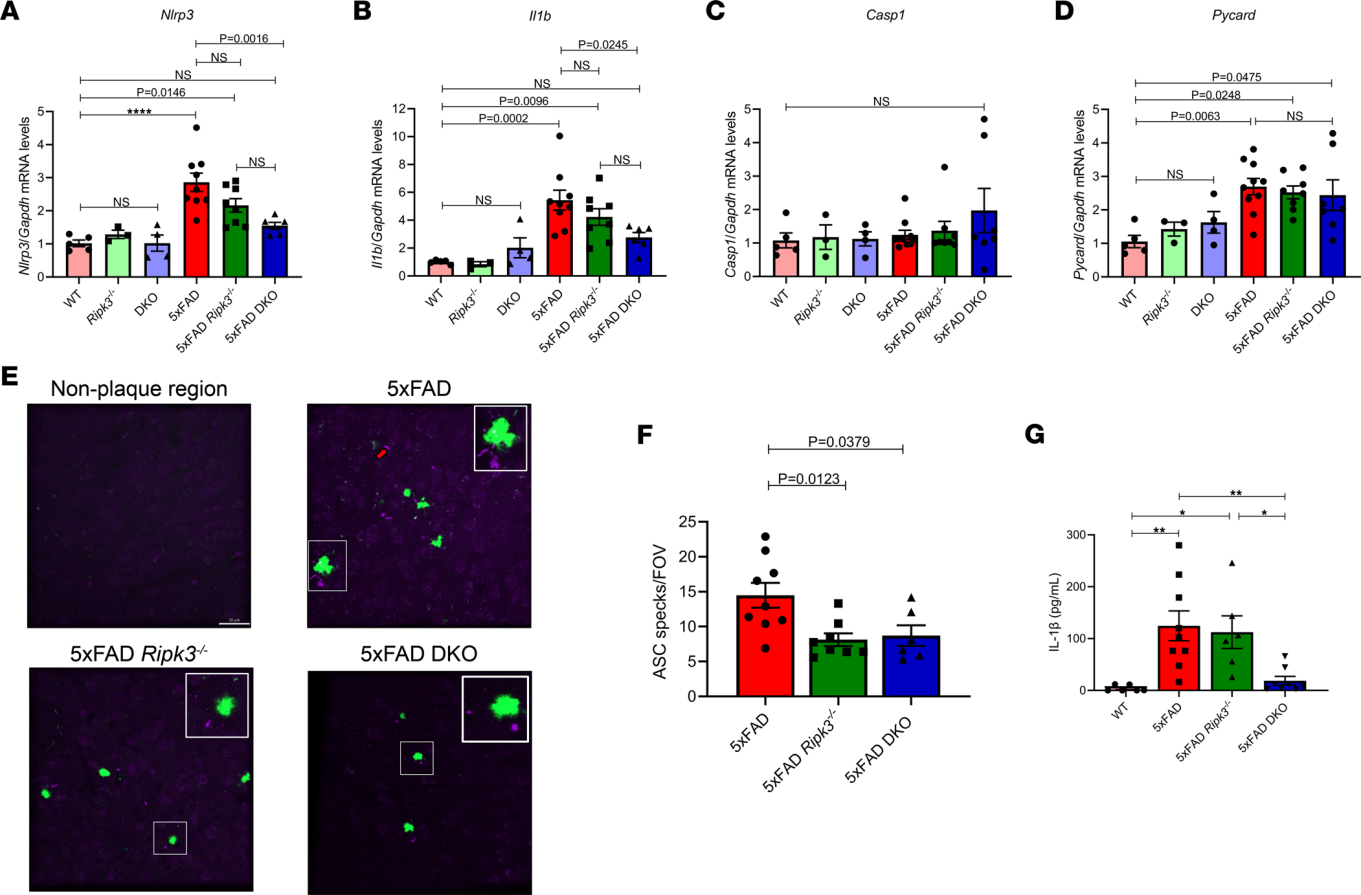
The caspase-8/RIPK3 axis regulates inflammasome signaling in 5xFAD mice. (**A**–**D**) *Nlrp3* gene transcript levels (**A**), *Il1b* gene transcript levels (**B**), *Casp1* gene transcript levels (**C**), and *Pycard* gene transcript levels (**D**) from the hippocampi of 5-month-old mice (*n* = 5 WT mice, *n* = 3 *Ripk3^–/–^* mice, *n* = 4 DKO mice, *n* = 9 5xFAD mice, *n* = 8 5xFAD *Ripk3^–/–^* mice, and *n* = 6 5xFAD-DKO mice). ****P < 0.0001. (**E**) Representative IHC images of the cortex of 5-month-old mice, stained for ThioS (green) and ASC (purple), taken at ×40 original magnification (scale bar: 30 μm; *n* = 9 5xFAD mice, *n* = 8 5xFAD *Ripk3^–/–^* mice, and *n* = 6 5xFAD-DKO mice). Red arrow denotes ASC speck. (**F**) Quantification of the number of ASC specks per field of view (FOV) from **E**. (**G**) Supernatants were collected from ex vivo brain suspensions from 5-month-old mice after a 48-hour incubation and evaluated for IL-1β release via ELISA: WT versus 5xFAD, **P = 0.0050; WT versus 5xFAD *Ripk3^–/–^*, *P = 0.0247; 5xFAD versus 5xFAD DKO, **P = 0.0077; 5xFAD *Ripk3^–/–^* versus 5xFAD DKO, *P = 0.0418 (*n* = 6 WT mice, *n* = 9 5xFAD mice, *n* = 6 5xFAD *Ripk3^–/–^* mice, and *n* = 8 5xFAD-DKO mice). Data were analyzed by 1-way ANOVA followed by Tukey’s post hoc test. Data are expressed as mean ± SEM.

The above in vitro studies pointed to a potential role for caspase-8 in regulating NLRP3 inflammasome priming after Aβ challenge. To validate our findings in vivo, we assessed transcript levels for *Nlrp3*, *Il1b*, *Casp1*, and *Pycard* in hippocampal lysates. We observed that Aβ deposition in 5xFAD mice led to elevated levels of *Nlrp3* and *Il1b*, in comparison with levels in WT controls (Figure 7, A and B). Moreover, we found that combined deletion of caspase-8 and RIPK3, but not RIPK3 alone, in 5xFAD mice reduced *Nlrp3* and *Il1b* transcript expression to WT levels (Figure 7, A and B). In contrast, no changes in *Nlrp3* and *Il1b* expression were detected with targeted deletion of the caspase-8/RIPK3 axis in non-5xFAD mice (Figure 7, A and B). We also did not observe any changes to the *Casp1* transcript (Figure 7C). Interestingly, *Pycard* expression was upregulated in 5xFAD transgenic mice, but loss of caspase-8 and/or RIPK3 did not alter its levels (Figure 7D). Overall, these results point to a role for caspase-8 in regulating transcription of inflammasome components in the 5xFAD model.

During inflammasome assembly, ASC forms fibril-like structures that can be released into the extracellular space. It is thought that ASC speck release is important for sustaining an ongoing immune response (43, 44). More recently, it has been demonstrated that extracellular ASC specks can promote both the seeding and spread of Aβ in a prion-like fashion (31, 45). We were interested in examining ASC speck numbers across our genotypes. Upon staining for ASC, we observed ASC fibrils near plaques and rarely in non–plaque-containing regions. Interestingly, we found that loss of RIPK3 or combined loss of caspase-8 and RIPK3 in 5xFAD mice both led to a significant reduction in the number of ASC specks surrounding plaques (Figure 7, E and F).

To further characterize the role of the caspase-8/RIPK3 axis in regulating IL-1β secretion, we leveraged a previously described assay to measure IL-1β release from isolated brain cells ex vivo (46). Single-cell suspensions derived from cortical homogenates from WT, 5xFAD, 5xFAD *Ripk3^–/–^*, and 5xFAD-DKO mice were plated for 48 hours, and supernatants were collected for IL-1β ELISAs. We found that suspensions generated from 5xFAD and 5xFAD *Ripk3^–/–^* cortices released appreciable amounts of IL-1β. Consistent with our data in Figure 5B, combined loss of caspase-8 and RIPK3 led to a significant inhibition of IL-1β release (Figure 7G). Taken together, these data suggest that caspase-8 plays a central role in NLRP3 inflammasome signaling.

## Discussion

Cell death and inflammatory pathways play central roles in the pathogenesis and spread of AD. Protein aggregates such as Aβ and tau can act as damage-associated molecular patterns, giving rise to an immune response (1). Although acute inflammation is beneficial and can aid in Aβ phagocytosis, the chronic nature of the AD immune response is thought to incite further Aβ deposition, generating a pathological positive feedback loop. Identification of the molecular players responsible for driving AD-associated neuroinflammatory responses versus those that facilitate beneficial clearance of neurotoxic material, therefore, is vital.

Here, we show that combined deletion of caspase-8 and RIPK3 limited AD pathology. In contrast, we found that RIPK3 deletion alone did not appear to have an appreciable effect on pathology in the 5xFAD model, suggesting that many of the phenotypes observed in our 5xFAD-DKO mice are likely driven by caspase-8. Overall, these data suggest that the caspase-8/RIPK3 axis, and caspase-8 in particular, is critically involved in AD-related disease progression as measured by amyloid deposition and microgliosis in the 5xFAD model. Deposition in the 5xFAD model begins in the subiculum before spreading throughout the hippocampus and overlying cortex. Interestingly, we found that D54D2 deposition was unchanged in the subiculum at both 3 and 5 months, whereas ThioS^+^ plaques were significantly reduced in 5xFAD-DKO mice (Supplemental Figure 1). On the basis of this finding, it is tempting to speculate that caspase-8 may be more important for disease spread as opposed to initiation. In our analysis of D54D2^+^ Aβ plaques, we observed that 5xFAD-DKO mice had significantly fewer Aβ deposits that were small (0–10 μm^2^) in comparison with those of 5xFAD or 5xFAD *Ripk3^–/–^* mice (Figure 2G). Amyloid deposits become larger over time, with smaller plaques serving as seeds for larger deposits (47, 48). Therefore, our observations in 5xFAD-DKO mice point toward a potential role for caspase-8 in promoting plaque seeding and overall Aβ spread.

Caspase-8 is critical in mediating cell death and inflammatory responses in macrophage populations (24, 25, 49). Here, we demonstrate that combined deletion of caspase-8 and RIPK3 led to a significant dampening of the microglial response in the 5xFAD model. Both overall cortical microglial coverage and the microglial plaque response were reduced in 5xFAD-DKO mice. Given that 5xFAD-DKO mice also exhibited diminished plaque burden, it would appear that the reduced microglial activation/plaque interaction may be beneficial in this setting in contrast to other cases, such as with TREM2 loss, which leads to increased plaque load and worsening of cognitive function (37, 38). We suspect that caspase-8 plays a key role in regulating microglial responses to Aβ. Indeed, our morphological analysis revealed that 5xFAD-DKO mice have soma-to-branch volume ratios that are similar to those of their nontransgenic counterparts. This reduction in microglial activation likely contributes to the reductions in Aβ aggregates that we observed in our 5xFAD-DKO mice. Although it is likely that the smaller number of Aβ aggregates contributes to the diminished microglial reactivity in 5xFAD-DKO mice, we believe that changes in overall amyloid burden are probably driven by intrinsic changes in microglial function. This reasoning is supported by our findings that loss of caspase-8 and RIPK3 led to reductions in the number of microglia surrounding individual plaques, our morphological analysis showing diminished activation in 5xFAD DKO microglia, and work from Spangenberg et al. (50) in which they demonstrated that depletion of microglia in the 5xFAD model leads to marked reductions in parenchymal plaque deposition. Overall, our work positions caspase-8 as a key regulator of amyloidosis and Aβ-mediated microgliosis.

We observed that loss of RIPK3 did not significantly alter amyloid deposition and gliosis in 5xFAD mice, which, despite components of the necroptotic signaling pathway being reportedly activated in AD (15), argues for RIPK3 not appearing to appreciably affect Aβ amyloidosis in 5xFAD mice. However, in many of our studies, we were only able to detect significant changes when comparing 5xFAD mice with their 5xFAD-DKO counterparts and not between 5xFAD *Ripk3^–/–^* and 5xFAD-DKO mice. This finding may point to a partial protective effect of RIPK3 deletion in the 5xFAD model. A number of groups have demonstrated a role for RIPK3 in ERK- and NF-κB–mediated cytokine production that occurs either prior to or independent of cell death (51, 52). Although we largely observed significant protection against amyloid deposition and microgliosis with combined deletion of both caspase-8 and RIPK3, it is still possible that RIPK3 affects other aspects of disease pathogenesis that were not specifically explored in our studies.

Moreover, it should be noted that the possibility still exists that RIPK3 and necroptosis could still play more significant roles during the later stages of AD, as other groups have suggested (15). Many previous studies relied on the targeting of RIPK1, which is best known as an upstream regulator of RIPK3 but can also initiate noncanonical inflammatory pathways and other forms of cell death that are independent of RIPK3 and necroptosis (26, 53–56). In these previously published studies, it was shown that inhibiting RIPK1 limits neurodegenerative disease (54–56). Coupling these published RIPK1 findings with those of our studies demonstrating that RIPK3 deletion does not significantly affect Aβ amyloidosis suggests that RIPK1 likely contributes to neurodegenerative disease through its regulation of noncanonical inflammatory cytokine production and not via induction of RIPK3-dependent events. Consistent with this idea, a recent study revealed a cell death–independent role for RIPK1 in the regulation of microglial responses in the APP/PS1 mouse model of AD (54). More specifically, the authors showed that RIPK1 coordinates the acquisition of the DAM phenotype in APP/PS1 mice.

Caspase-8 is an initiator caspase in the extrinsic apoptotic pathway. It is possible that alterations in cell death execution resulting from loss of caspase-8 and/or RIPK3 could be contributing to the observed phenotypes. Consistent with previous reports, we also observed a reduction in cortical layer V neuron density when comparing 5xFAD and WT mice (40, 41). However, we observed no differences in NeuN^+^ density between 5xFAD, 5xFAD *Ripk3^–/–^*, and 5xFAD-DKO mice. Taken together, these data suggest that caspase-8 is required for AD progression; however, to our surprise, it does not appear that caspase-8 appreciably affects neuronal cell death in 5xFAD mice, despite playing a substantial role in promoting amyloidosis and microgliosis. This observation suggests that the cell loss that occurs in response to Aβ amyloidosis in 5xFAD mice is possibly mediated by caspase-8–independent death pathways, such as intrinsic apoptosis, pyroptosis, and ferroptosis, as others have illustrated (57–59).

AD pathology spreads across the brain over the course of decades. Deposition of tau and Aβ follows a fairly stereotyped pattern of progression, with initial signs emerging in the transentorhinal cortex before proceeding to higher order cortical regions (60, 61). Oligomeric forms of Aβ, when harvested from patient samples, can act as seeds and promote pathology when injected into the hippocampus of presymptomatic mice (62). In a similar prion-like fashion, pathogenic tau harvested from patients can seed endogenous mouse tau and propagate spread (63). Several studies have demonstrated evidence for both transsynaptic and nonsynaptic modes of disease progression (63–66). For example, microglia can release extracellular vesicles containing tau aggregates, which contribute significantly to nonsynaptic spread of pathology (64). In addition, several studies have implicated members of both the TNFRSF and TLR family in driving neuronal loss, inflammation, and disease spread in AD. Interestingly, caspase-8 serves as a common downstream signaling mediator for many of these TNFRSF and TLR receptors (19).

How precisely might caspase-8 influence the spread of AD pathology? Protein aggregates such as Aβ and tau can activate the NLRP3 inflammasome (28, 29, 31), the components of which are predominantly expressed by astrocytes and microglia in the CNS (67, 68). We observed in 5xFAD mice elevated hippocampal transcript levels of both *Nlrp3* and *Il1b*, which were significantly dampened with co-deletion of caspase-8 and RIPK3 but not RIPK3 alone. A by-product of inflammasome activation is the release of ASC to the extracellular space. A number of studies have shown that ASC specks can seed Aβ and tau deposits (29, 31, 45). Thus, inhibition of ASC or upstream inflammasome components can dampen both amyloid and tau aggregation. Indeed, 5xFAD-DKO mice had significantly fewer ASC specks in plaque regions than did their 5xFAD counterparts. Interestingly, loss of RIPK3 also led to a significant reduction in ASC specks (Figure 7, E and F). Our findings that loss of RIPK3 was insufficient to significantly diminish amyloid load suggest that ASC specks may only be part of the equation. IL-1β has been documented as being a critical regulator of microglial proliferation and activation (69). Our ex vivo assay revealed that cortical single-cell suspensions from 5xFAD-DKO mice produced significantly less IL-1β than suspensions from 5xFAD and 5xFAD *Ripk3^–/–^* mice (Figure 7G). Previous reports have suggested that events downstream of NLRP3 inflammasome activation, such as IL-1β release and speck generation, are not necessarily coupled (70). It is possible caspase-8’s combined regulation of gliosis, NLRP3 inflammasome component expression, IL-1β release, and ASC speck generation are collectively required to delay amyloid spread in 5xFAD mice. Moreover, it is likely that caspase-8 is governing other aspects of AD progression that we did not explore here. Several studies have demonstrated that caspase-8 is a key regulator of inflammatory gene expression in large part through NF-κB signaling (19, 22). We examined phospho–NF-κB levels from hippocampal lysates but observed no differences across our transgenic mice (data not shown). However, it is possible that there are subtle changes to NF-κB that are occurring in specific cell populations (e.g., microglia) that we did not capture in our bulk analysis. Therefore, it will be interesting to fully examine the scope of how caspase-8 regulates the AD inflammatory profile.

Overall, the work presented here provides insights into mechanisms governing AD progression. We demonstrate that the caspase-8/RIPK3 axis is critical for promoting both Aβ deposition and gliosis in the 5xFAD mouse model of AD. Furthermore, we show that combined deletion of caspase-8 and RIPK3 limits both the priming of the NLRP3 inflammasome and IL-1β secretion in response to Aβ. This work helps establish caspase-8 as a novel mediator of AD spread and suggests that caspase-8 may be a potential target to mitigate AD pathology.

## Methods

### Mice.

5xFAD mice were purchased from The Jackson Laboratory (catalog 34848) and were crossed with either Ripk3 or Caspase-8 Ripk3-DKO mice to generate 5xFAD-DKO mice (13, 18, 71). Animals were housed on a 12-hour light/12-hour dark cycle. Primers used for genotyping were 5xFAD (5′-CGGGCCTCTTCGCTATTAC-3′, 5′-TATACAACCTTGGGGGATGG-3′, 5′-ACCCCCATGTCAGAGTTCCT-3′), caspase-8 (5′-GGATGTCCAGGAAAAGATTTGTGTC-3′, 5′-CCTTCCTGAGTACTGTCACCTGT-3′), and RIPK3 (5′-CGCTTTAGAAGCCTTCAGGTTGAC-3′, 5′-GCAGGCTCTGGTGACAAGATTCATGG-3′, 5′-CCAGAGGCCACTTGTGTAGCG-3′) (Integrated DNA Technologies). Non-5xFAD transgenic littermates were used as controls during the study. All mice used in experiments were male.

### Cell culture.

Briefly, mixed glial cultures were prepared from the cortices of newborn mice (P0–P2). Meninges were carefully removed in ice-cold DMEM/F12 (Thermo Fisher Scientific) and cortices dissociated via trituration. Dissociated cortices were spun at 600*g* for 5 minutes. Cells from 2 brains (*n* = 4 cortices) were plated in a T-75 flask coated with poly-d-lysine (Sigma) in 15 mL of DMEM/F12 (Thermo Fisher Scientific) with 10% FBS (Gibco), 1% penicillin and streptomycin (Thermo Fisher Scientific), sodium pyruvate (Thermo Fisher Scientific), and MEM Non-Essential Amino Acids (Thermo Fisher Scientific). On day in vitro 7 (DIV7) and DIV9, 5 mL of L-929 cell–conditioned medium (LCM) was added to promote microglial growth. On DIV12, glial cultures were trypsinized and plated at 100,000 cells/well of a 24-well plate. Cells were treated on DIV2.

### Cell treatments.

For IL-1β release studies, cells were primed with 500 ng/mL LPS (Sigma, catalog L4391) for 3 hours, then treated with oligomeric Aβ (oAβ) for 24 hours. Supernatants were collected and analyzed for IL-1β (Thermo Fisher Scientific, catalog 88-7013-22) according to manufacturer instructions.

### Preparation of LCM.

L-929 cells (ATCC) were cultured in a T-25 flask with 5 mL of DMEM (Thermo Fisher Scientific) with 10% FBS (heat-inactivated), 1% HEPES, 1% l-glutamine, and sodium pyruvate. Upon reaching confluence, cells were lifted with 1× Trypsin-EDTA (Thermo Fisher Scientific), resuspended in 15 mL of medium, transferred to a T-75 flask. After reaching confluence, cells were again lifted with Trypsin-EDTA and transferred to a T-175 flask in 50 mL of medium. After 7 days in culture, the supernatant was harvested and spun at 1,700*g* for 5 minutes, then filtered through a 0.45 μm filter before freezing at –80°C.

### Preparation of oAβ.

oAβ was prepared as described (72). We dissolved 1 mg of Aβ_1-42_ peptides (Echelon Biosciences) in 250 μL of 1,1,1,3,3,3-hexafluoro-2-propanol (HFIP; Sigma-Aldrich) via room temperature incubation for 2–3 hours. Upon dissolution, Aβ peptide was aliquoted into Eppendorf tubes. HFIP was evaporated overnight in a biosafety cabinet, and the dried peptide was stored at –80°C. Before use, aliquots were kept at room temperature for 15 minutes. Films were dissolved in anhydrous DMSO (Sigma) to 5 mM. Cold phenol-free F-12 medium (Thomas Scientific, catalog C994K53) was added to yield a final concentration of 100 μM. The resulting solution was then vortexed for 15 seconds and left at 4°C for 24 hours with gentle rocking.

### Tissue preparation.

Mice were euthanized via CO_2_ inhalation and transcardially perfused with 50 mL of ice-cold PBS. Brains were removed from the skull. One hemisphere was fixed in 4% paraformaldehyde (PFA) for 24 hours while the other was micro-dissected into cortex and hippocampus and frozen in dry ice and then transferred to –80°C.

### IHC.

PFA-fixed hemispheres were washed with PBS (3 washes, 10 minutes per wash) then transferred to 30% sucrose/PBS for 48–72 hours. Brains were then frozen on dry ice after they sank. Free-floating, 30 μm thick serial coronal sections were collected on a cryostat and kept in 1× PBS plus 0.002% sodium azide at 4°C. Sections were washed 3 times for 5 minutes in PBS, blocked in 2% normal donkey serum plus 1% BSA plus 0.1% Triton plus 0.05% Tween-20 for 1 hour, incubated overnight at 4°C in blocking solution and primary Ab (Iba1, goat, 1:500, Abcam, catalog ab5076; GFAP, mouse, 1:500, EMD Millipore, catalog MAB360; D54D2, rabbit, 1:500, Cell Signaling Technology, catalog 8243S; CD68, rat, 1:800, BioRad, catalog MCA1957; ASC, rabbit, 1:300, Adipogen, catalog AL177; NeuN, rabbit, 1:1,000, Abcam, catalog ab177487; Dectin-1 (Clec7A), 1:30, InvivoGen, catalog mabg-mdect; and Ctip2, 1:400, Abcam, catalog ab240636), washed again, then incubated in blocking solution with the corresponding Alexa Fluor secondary Abs (1:500) — donkey anti-rabbit IgG (H+L) Alexa Fluor 568, A10042 (Thermo Fisher Scientific), donkey anti-mouse IgG (H+L) Alexa Fluor 568, A10037 (Thermo Fisher Scientific), donkey anti-goat IgG (H+L) Alexa Fluor 647, A-21447 (Thermo Fisher Scientific), and donkey anti-rat IgG (H&L) Alexa Fluor 647 preadsorbed, ab150155 (Abcam) — for 2 hours in darkness at room temperature, washed again, and then mounted in Fluoromount-G (SouthernBiotech, catalog 0100-01).

For ThioS staining, after secondary Ab–staining incubation and washes, sections were incubated in 1% ThioS in 80% ethanol for 15 minutes at room temperature. Sections were then washed for 2 minutes in 80% ethanol, 70% ethanol, and deionized water 2 times, then mounted using Fluoromount G. TUNEL labeling was carried out using the In Situ Cell Death Detection Kit, Fluorescein (Roche), according to manufacturer’s instructions, after Iba1 secondary staining and washes.

### ISH assay by RNAscope.

Brains were dissected from C57BL/6N and 5xFAD mice and fixed with 4% PFA in PBS for 24 hours at 4°C. Tissues were then incubated in 30% sucrose solution at 4°C overnight. Fixed tissues were then frozen on dry ice and stored at –80°C. Frozen tissues were cryosectioned at 20 μm thickness, collected onto positively charged slides (Shandon Superfrost Plus, Thermo Fisher Scientific, catalog 6776214), and air-dried overnight in the dark. The following day, slides were washed with PBS (2 washes for 2 minutes per wash) then incubated for 10 minutes in H_2_O_2_ (RNAscope H_2_O_2_ and Protease Reagents Kit, Advanced Cell Diagnostics, catalog 322381). Slides were then twice washed for 2 minutes with distilled water to remove H_2_O_2_ before incubation with protease IV solution (RNAscope H_2_O_2_ and Protease Reagents Kit, Advanced Cell Diagnostics, catalog 322381) for 30 minutes at 40°C in a HybEZ II oven (Advanced Cell Diagnostics, catalog 321710/321720) and 2 additional 2-minute washes in distilled water. Tissues were then incubated in Probe Master Mix for 2 hours at 40°C (Probe1: Mm-RipK3, catalog 462541; Probe2: Mm-Casp8-C2, catalog 468971-C2; Advanced Cell Diagnostics) and then washed twice in 1× wash buffer (RNAscope Wash Buffer Reagents, Advanced Cell Diagnostics, catalog 310091).

After amplification by sequential incubations with AMP1, 2, and 3 solutions (RNAscope Multiplex Fluorescent Detection Kit v2, Advanced Cell Diagnostics, catalog 323110) for 30 minutes at 40°C (separated with two 2-minute washes with wash buffer between solutions), slides were incubated for 15 minutes at 40°C with HRP-Channel 1 (RNAscope Multiplex Fluorescent Detection Kit v2, Advanced Cell Diagnostics, catalog 323110), before an additional 2 washes for 2 minutes in wash buffer. Slides were then incubated in a fluorescent dye for 30 minutes at 40°C (1:750 dilution; Tyramide Signal Amplification [TSA] Cyanine 3, Akoya, catalog TS000202) before two 2-minute washes with wash buffer and blocking with HRP blocker for 15 minutes at 40°C. The same HRP steps were repeated for channel 2 by applying a second fluorescent dye (1:750 dilution; TSA Fluorescein, Akoya; catalog TS000200). Finally, 10–20 μL of DAPI was applied at room temperature to stain the nuclei (DAPI Fluoromount-G, SouthernBiotech, catalog 0100-20), and then slides were sealed with coverslips.

### Image analysis.

Images were taken using a Zeiss 980 confocal laser-scanning microscope at either ×10/.45, ×20/0.8, or ×40/1.3 (oil) original magnification. Maximum intensity projections of *Z*-stacks were used for all analysis. For percentage area measurements and plaque counts, 8-bit images were exported to FIJI. To analyze D54D2 staining, regions of interest covering the RS or subiculum were drawn, and analysis was performed following background subtraction (rolling-ball radius, 50 pixels) and thresholding using the Intermodes function. ThioS^+^ plaques were manually counted in both the RS and subiculum. For Iba1 and GFAP percent area measurements, 8–10 fields of view throughout the cortex were taken across 2 sections at ×40 original magnification. For Iba1 staining, analysis was performed after background subtraction (rolling-ball radius, 50 pixels), applying an unsharp mask filter (3.0, 0.6), despeckle, and thresholding (min intensity 32, max intensity 255). GFAP analysis was performed after background subtraction (rolling-ball radius, 50 pixels) and thresholding (min intensity 54, max intensity 255).

Images for both microglial periplaque and Sholl analyses were taken at ×40 original magnification. Local microgliosis was evaluated for ThioS^+^ plaques spanning the cortex. Plaques were excluded if they were within 15 μm of another plaque. Images were imported into Imaris 9.7.2 and plaques and were labeled using the Surfaces feature. For volume-fraction calculations, microglia were labeled using the Surfaces feature and the volume of Iba1 staining within 15 μm of each plaque was calculated. For Iba1^+^ cell counts, microglia were labeled using the Spots feature, and microglia within 15 μm of each plaque were counted. In total, 46–51 microglia were analyzed per experimental group (*n* = 6 mice/group). Sholl analysis was performed using the filament tracer tool in Imaris 9.7.2. Microglial soma volumes were determined using the Surfaces feature in Imaris. Microglia were excluded if they were within 15 μm of a plaque and/or the entire microglia was not covered in the field of view. For each mouse, 10–15 cortical microglia were chosen for a total of 70–80 microglia/group (*n* = 6 mice/group).

For ASC speck quantification, images were taken at ×40 original magnification. Random plaque-containing fields of view were taken across the cortex. ASC specks were counted within 15 μm of plaques.

For NeuN analysis, images from cortical layer V were taken at ×20 original magnification, and NeuN^+^ cells were counted in FIJI. Cells were counted using the Analyze Particles feature (greater than 12 μm^2^) after thresholding (min intensity 116, max intensity 255) and watershed separation.

For RNAscope analysis, images from the subiculum, cortex, and thalamus were taken at ×40 original magnification. *Casp8*^+^ and *Ripk3^+^* puncta were counted in FIJI using the Analyze Particles feature (greater than 0.8 μm^2^) after thresholding.

### ELISA quantification of Aβ.

Snap-frozen cortices were massed and homogenized initially in 1× RIPA buffer (25 mM Tris-HCl, pH 7.5, 150 mM NaCl, 1% NP-40, 0.5% sodium deoxycholate, 0.1% SDS) supplemented with complete protease inhibitor (Roche) and PhosSTOP phosphatase inhibitor (Roche). Homogenates were spun at 16,000*g* for 20 minutes, and the supernatants were collected and saved at –80°C as the RIPA-soluble fraction. Pellets were further homogenized in ice-cold 5 M guanidine–HCl diluted in 50 mM Tris at pH 8.0. Homogenates were mixed on a shaker for 3–4 hours at room temperature, then stored in –80°C as the guanidine-soluble fraction. ELISAs for Aβ_1-42_ were run using commercial kits (R&D Systems).

### Real-time PCR.

Total RNA was extracted from hippocampal tissue using Trizol (Thermo Fisher Scientific, catalog 15596026) combined with the RNeasy Mini Kit (Qiagen, catalog 74104). For cDNA synthesis, 500 ng of RNA was used (Thermo Fisher Scientific, catalog 18080051). Transcript levels of *Nlrp3* (Thermo Fisher Scientific, assay ID Mm00840904_m1), *Il1b* (Thermo Fisher Scientific, assay ID Mm00434228_m1), *Casp1* (Thermo Fisher Scientific, assay ID Mm00438023_m1), and *Pycard* (Thermo Fisher Scientific, assay ID Mm00445747_g1) were quantified by RT-PCR using a CFX96 Real-Time System. *Gapdh* (Thermo Fisher Scientific, assay ID Mm99999915_g1) expression was used for normalization, and results are presented as a fold change induction over levels in WT samples.

### Ex vivo IL-1β release assay.

Cortical suspensions were prepared as described previously (46), with some modifications. Briefly, cortices were minced and harvested in HBSS (with calcium and magnesium). HBSS was then aspirated and replaced with enzyme solution (2 mg/mL papain, 1 mg/mL DNase I in HBSS with calcium and magnesium). Cortices were passed through an 18G needle, then incubated for 45 minutes at 37°C for digestion. To aid in the dissociation, tissue was removed every 15 minutes and passed through an 18G needle. After incubation, tissue was passed through a 70 μm filter, then washed with complete RPMI (cRPMI) medium (10% FBS, 1% penicillin/streptomycin, 1% sodium pyruvate, 1% nonessential amino acids, and 0.1% 2-ME). Tissue was then resuspended in 10 mL of 40% percoll and spun at 650*g* for 25 minutes (no brake, 22°C). Supernatant was aspirated and the pellet resuspended in cRPMI and then spun at 650*g* for 5 minutes to remove residual percoll (full brake, 22°C). Pellet was resuspended in cRPMI and cells were plated at 100,000/well of a 96-well plate. After 48 hours, supernatants were collected and evaluated by ELISA for IL-1β release.

### Statistics.

Data in figures represent mean ± SEM. Each *n* represents an independent biological sample. Analysis was performed with GraphPad Prism, version 8.0, applying either a 1- or 2-way ANOVA with Tukey’s post hoc test comparing all groups with each other. All Student’s *t* tests were 2 tailed. Statistical significance is defined as *P* < 0.05.

### Study approval.

All experiments were carried out in compliance with the Association for Assessment of Laboratory Animal Care policies and approved by the University of Virginia Animal Care and Use Committee.

## Author contributions

SK, CDD, and JRL designed all experiments. SK conducted all experiments with assistance from SB, CBO, and ADK for mouse genotyping, tissue harvesting, preparation of glial cultures, and image analysis. OYC carried out the RNAscope staining. SK acquired and interpreted all data. SK, CDD, and JRL prepared figures and wrote the manuscript with input from all the authors.

## Supplementary Material

Supplemental data

## Version 1 01/05/2023

In-Press Preview

## Version 2 02/08/2023

Electronic publication
