# Supplementary material for: Role of the caspase-8/RIPK3 axis in Alzheimer’s disease pathogenesis and Aβ-induced NLRP3 inflammasome activation
Source: JCI Insight. 2023 Feb 8;8(3):e157433. doi: 10.1172/jci.insight.157433 (PMC9977425; doi:10.1172/jci.insight.157433)
Supplement: Supplemental data [file jciinsight-8-157433-s064.pdf]

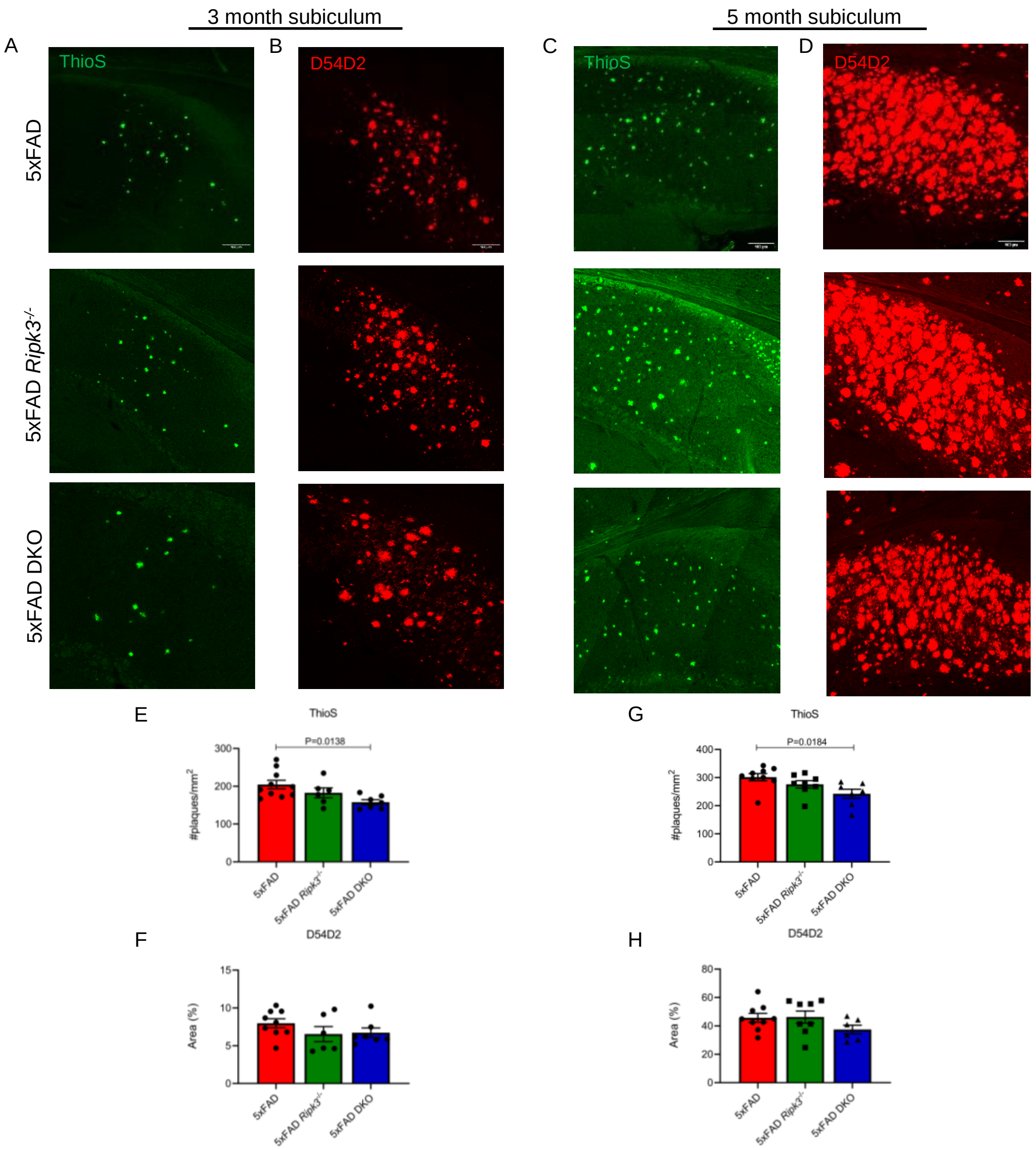

Supplemental Figure 1. The Caspase-8/RIPK3 axis regulates subicular amyloid deposition. Representative IHC images of the subiculum taken at 40x magnification (n=10 for 5xFAD, n=6 for 5xFAD *Ripk3*$^{-/-}$, and n=7 for 5xFAD DKO). (A) Representative ThioS staining at 3 months. (B) Representative D54D2 staining at 3 months. (C) Representative ThioS staining at 5 months. (D) Representative D54D2 staining at 5 months. (E-H) Quantification for (A-D). Data were analyzed by one-way ANOVA followed by Tukey post hoc test. All n values refer to the number of mice used and error bars indicate s.e.m.

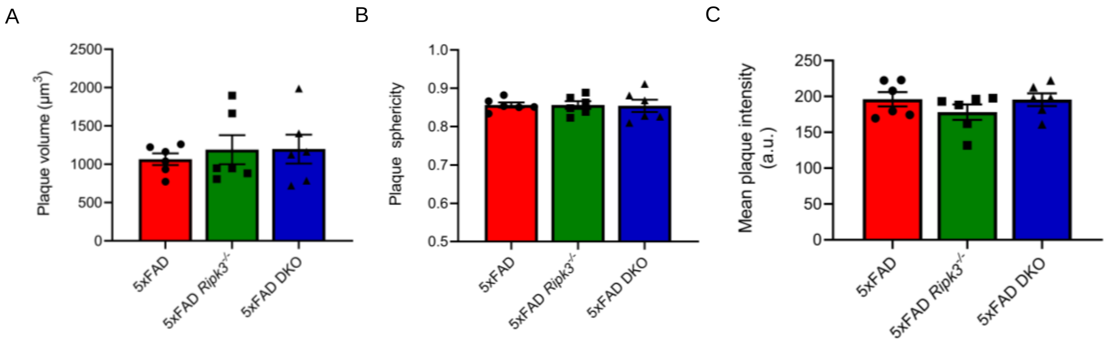

Supplemental Figure 2. Genetic ablation of Caspase-8 and/or RIPK3 in 5xFAD mice does not alter Aβ plaque sphericity, intensity, or volume. Cortical Thios$^+$ plaques were imaged at 40x magnification and labelled using the Surfaces feature in Imaris 9.7.2 and evaluated for (A) sphericity, (B) mean intensity, and (C) volume. (A-C) Results from 6-10 plaques were averaged per data point (n=6 for 5xFAD, n=6 for 5xFAD *Ripk3*$^{-/-}$, and n=6 for 5xFAD DKO). Data were analyzed by one-way ANOVA followed by Tukey post hoc test. All n values refer to the number of mice used and error bars indicate s.e.m.

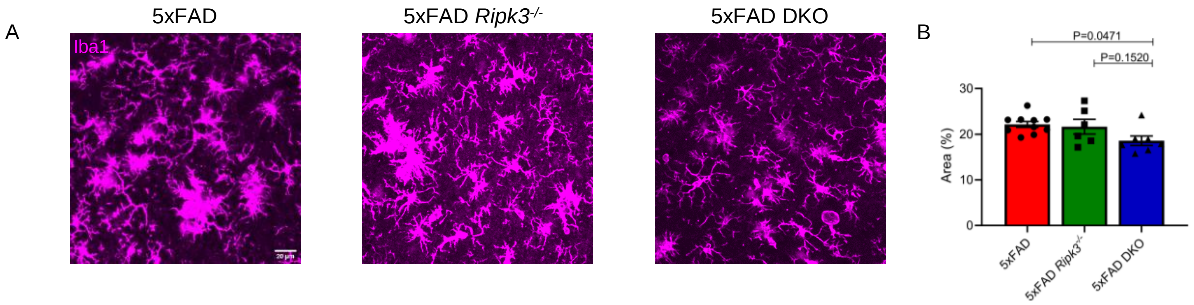

Supplemental Figure 3. Reduced subicular microgliosis with loss of Caspase-8 and RIPK3 in 5xFAD mice. Representative IHC images of the subiculum taken at 40x magnification for 3-month-old mice (n=10 for 5xFAD, n=6 for 5xFAD *Ripk3*$^{-/-}$, and n=7 for 5xFAD DKO). (A) Representative Iba1 staining. (B) Quantification of Iba1 staining. Each data point is an average of 2-3 different fields of view taken throughout the subiculum over 3 sections per mouse. Data were analyzed by one-way ANOVA followed by Tukey post hoc test. All n values refer to the number of mice used and error bars indicate s.e.m.

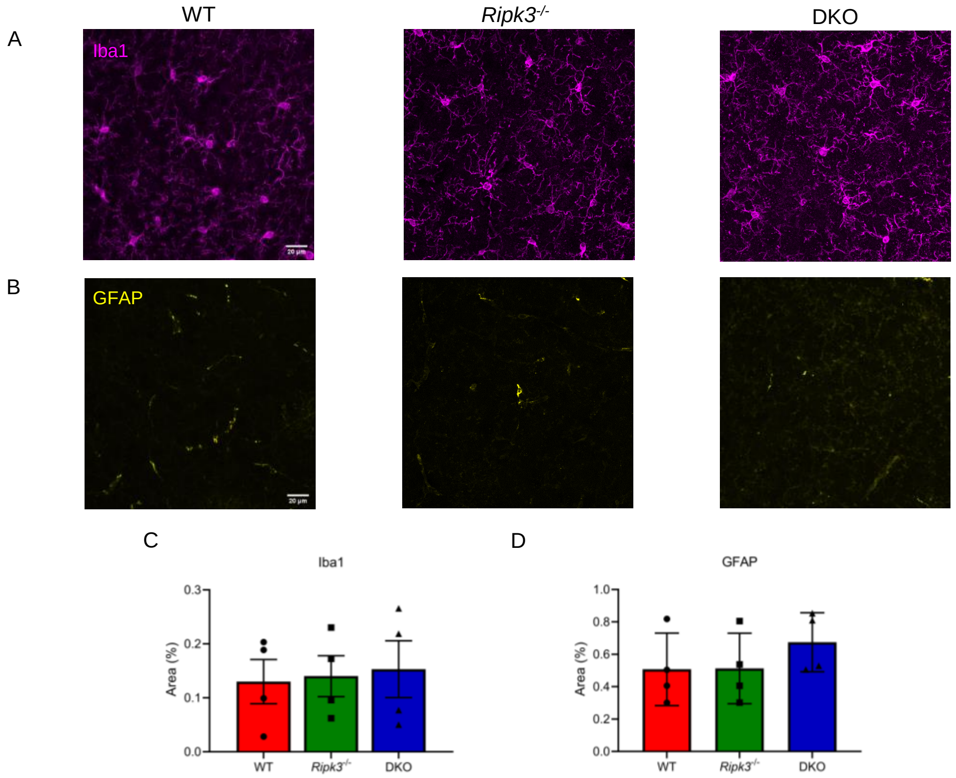

Supplemental Figure 4. Genetic deletion of Caspase-8 and/or RIPK3 does not affect Iba1 or GFAP staining in the absence of Aβ amyloidosis. Representative IHC images of the cortex from 5-month-old mice taken at 40x magnification (n=5 for WT, n=5 for *Ripk3*$^{-/-}$, and n=6 for DKO). (A) Representative Iba1 staining. (B) Representative GFAP staining. (C) Quantification of Iba1 staining. (D) Quantification of GFAP staining. (C-D) Each data point is an average of 8 different fields of view taken throughout the cortex over 2 sections per mouse. Data were analyzed by one-way ANOVA followed by Tukey post hoc test. All n values refer to the number of mice used and error bars indicate s.e.m.

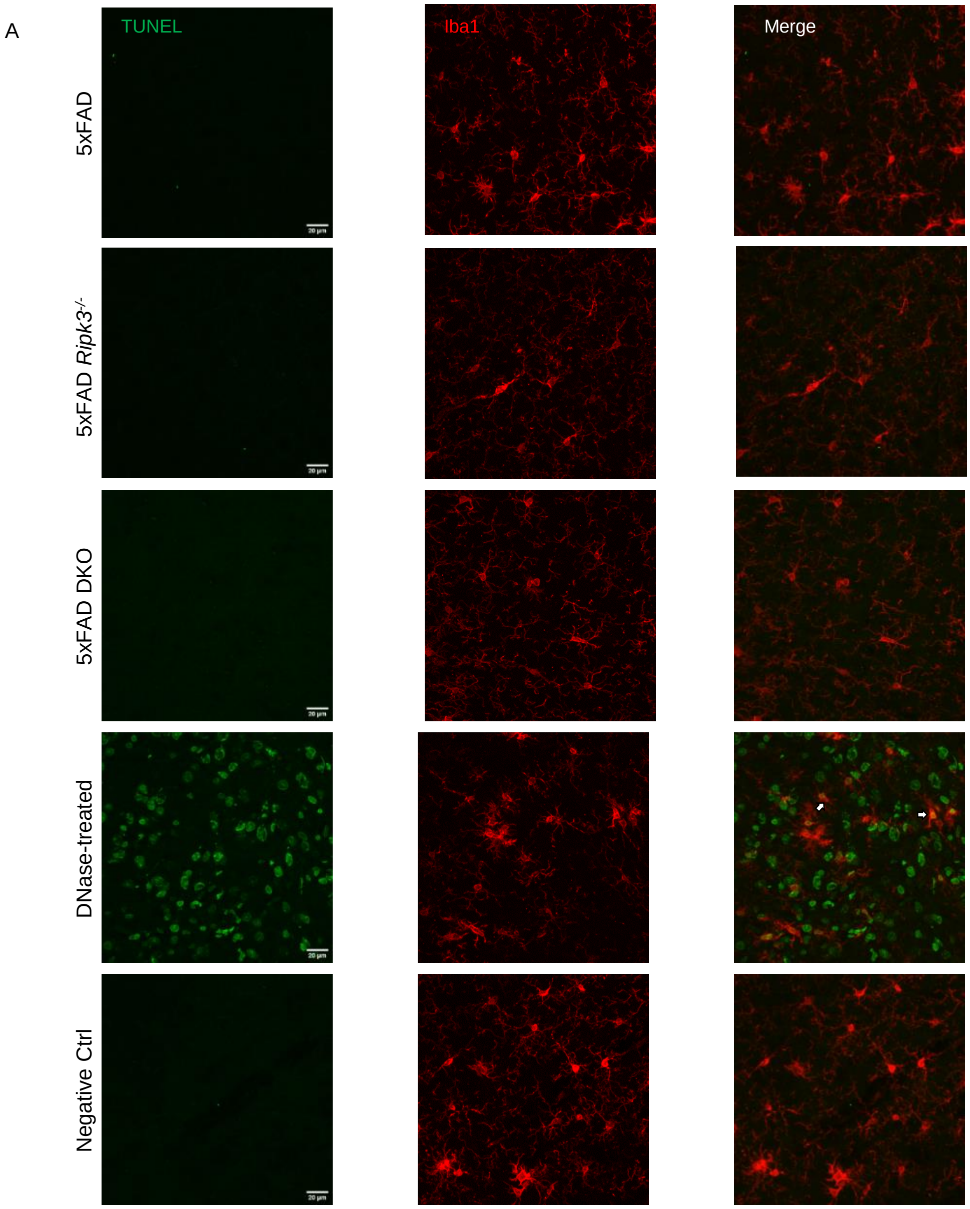

Supplemental Figure 5. Absence of TUNEL⁺ microglia in 5xFAD mice. (A) Representative cortical sections from 5xFAD, 5xFAD *Ripk3*$^{-/-}$, and 5xFAD DKO mice. DNase-treated and negative control cortical sections also included. White arrowheads indicate examples of TUNEL^{+} microglia in DNase-treated cortical sections.

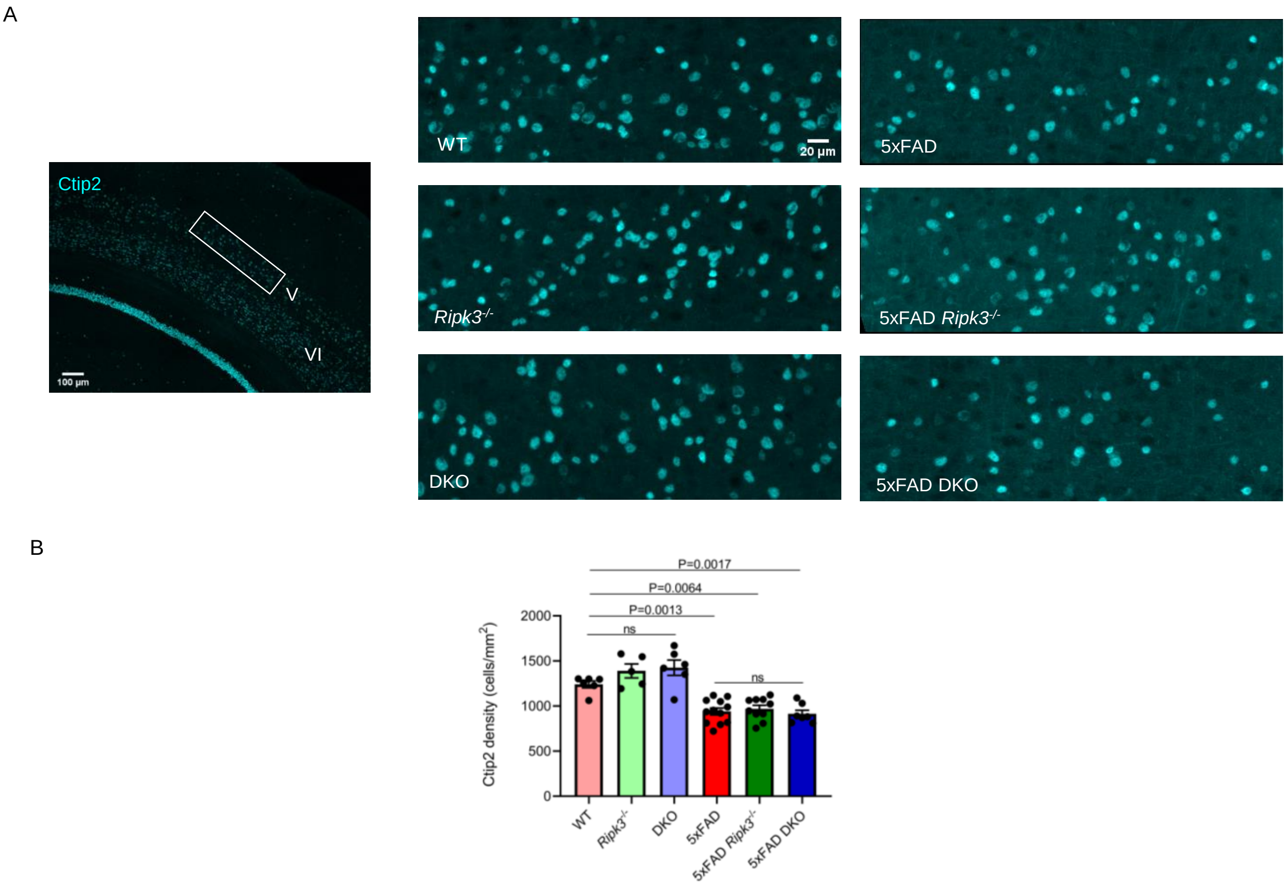

Supplemental Figure 6. Layer V identification via Ctip2. (A) Coronal sections were stained to mark cortical layers V and VI with white rectangle marking region of NeuN quantification (left). Representative IHC images of cortical layer V stained with Ctip2 taken at 20x magnification (right) (n=6 for WT, n=5 for *Ripk3*$^{-/-}$, n=6 for DKO, n=12 for 5xFAD, n=10 for 5xFAD *Ripk3*$^{-/-}$, and n=7 for 5xFAD DKO). (B) Quantification of Ctip2 staining. Data were analyzed by one-way ANOVA followed by Tukey post hoc test. Data expressed as mean ± s.e.m.
